# Effects of *Cordyceps militaris* fermentation products on reproductive development in juvenile male mice

**DOI:** 10.1038/s41598-022-18066-2

**Published:** 2022-08-12

**Authors:** Shan Lin, Wen-Kuang Hsu, Ming-Shiun Tsai, Tai-Hao Hsu, Tso-Ching Lin, Hong-Lin Su, Sue-Hong Wang, Dazhi Jin

**Affiliations:** 1 School of Laboratory Medicine and Bioengineering, Hangzhou Medical College, Hangzhou, 310053 Zhejiang China; 2 Key Laboratory of Biomarkers and In Vitro Diagnosis Translation of Zhejiang Province, Hangzhou, 311399 Zhejiang China; 3 Department of Medicinal Botanicals and Foods on Health Applications, Da-Yeh University, Changhua, 515006 Taiwan; 4 Department of Sport and Health Management, Da-Yeh University, Changhua, 515006 Taiwan; 5 Department of Life Sciences, National Chung Hsing University, Taichung, 402202 Taiwan; 6 Department of Biomedical Sciences, Chung Shan Medical University, Taichung, 402306 Taiwan; 7 Department of Medical Research, Chung Shan Medical University Hospital, Taichung, 402306 Taiwan

**Keywords:** Nutrition, Quality of life

## Abstract

*Cordyceps militaris* (CM) is a popular medicinal fungus; however, few studies have focused on its impact on the male reproductive system. We evaluated the effects of CM fermentation products on the reproductive development of juvenile male (JM) mice. Mice were divided into four experimental groups, each fed 5% CM products (weight per weight (w/w) in normal diet): extracellular polysaccharides (EPS), fermentation broth (FB), mycelia (MY), and whole fermentation products (FB plus MY, FBMY) for 28 days, while mice in the control group (CT) were fed a normal diet. Basic body parameters, testicular structure, sperm parameters, and sex hormones concentrations were analyzed. Compared to the CT group, mice in the EPS, MY, and FBMY groups showed a significantly increased mean seminiferous tubule area (*p* < 0.05), mice in the FB and MY groups had significantly higher sperm concentrations (*p* < 0.05), and mice in the EPS, FB, and FBMY groups showed significantly increased ratios of motile sperm (*p* < 0.05). Meanwhile, EPS significantly promoted the ability of JM mice to synthesize testosterone (*p* < 0.05). Furthermore, all CM products significantly increased the food intake of JM mice (*p* < 0.05) but did not significantly change their water intake and body weight gain (*p* > 0.05). In conclusion, CM products, especially EPS, exhibit strong androgen-like activities that can promote male reproductive development.

## Introduction

Testis development during juvenile stages is essential for male reproductive function^1^. Obesity^2^, environmental pollution^3^, psychological stress^4^, and even COVID-19^5^ impact testicular function and spermatogenesis. Exposure to these risk factors early in life may have long-term effects on male fertility^6^. A meta-analysis reported that sperm concentration has decreased globally by approximately 50% over the past 50 years, and male infertility has become a major problem for many couples^7^. The World Health Organization (WHO) predicts that infertility will become the third major health threat after cancer and cardiovascular diseases in the twenty-first century^8^. Currently, some drugs have been developed for treating male infertility^9^. For example, sildenafil citrate (Viagra) is used clinically to treat erectile dysfunction. However, many adverse events have been observed, including flushing, headaches, and dyspepsia^10^. Therefore, it is necessary to identify natural products that enhance male reproductive development and function without significant side effects.

*Ophiocordyceps sinensis* (*O. sinensis*), referred to as the “Himalayan Viagra,” is a scarce and expensive medicinal fungus^11^. *Cordyceps militaris* (*C. militaris*), also known as Northern *O. sinensis*, is a species of the genus *Cordyceps*^12^. Studies have shown that there are the levels of some bioactive ingredients, such as amino acids, unsaturated fatty acids, and adenosine, are higher in *C. militaris* than in *O. sinensis*^13^. Recently, *C. militaris* has been widely used as a substitute for *O. sinensis* in traditional Chinese medicine and health supplement^12^.

Specifically, the culture of *C. militaris* using a liquid fermentation process can shorten the production cycle and increase yield^14^. This process yields large amounts of extracellular polysaccharides (EPS) and mycelia (MY) in a short period. EPS and MY have been proven to possess antioxidant, antimicrobial, and anti-inflammatory activities^15,16^. However, due to the large volume of fermentation broth (FB) and low content of active ingredients, it is often discarded as waste. In this study, we investigated the effects of *C. militaris* fermentation products on the reproductive development of juvenile male (JM) mice. Basic body parameters, testicular structure, sperm parameters, and serum concentrations of sex hormones were analyzed.

## Results and discussion

### Main components of the four *C. militaris* fermentation products

Table 1 showed that the content of polysaccharides in the EPS group was the highest, and was significantly higher than those in the FB, MY and FBMY groups (*p* < 0.05). There were significant differences in the contents of cordycepin and adenosine in the four *C. militaris* fermentation products (*p* < 0.05), and all had the highest content in the FB. Except between the FB and EPS, protein concentrations in different fermentation products were all significant differences (*p* < 0.05). The protein concentrations in the MY was the highest, followed by in the FBMY, in the FB, and in the EPS was the lowest. In this study, EPS were obtained from FB by ethanol precipitation. When the EPS in FB were precipitated with ethanol, cordycepin could not be simultaneously precipitated because of its good solubility in water and ethanol. Thus, the cordycepin content in the EPS was below detectable levels.

**Table 1:** Main components of the four *C. militaris* fermentation products used in this study.

Samples	Yield (%)			
	Polysaccharides	Cordycepin	Adenosine	Proteins
EPS	12.20 ± 1.34^a^	N.D.*	0.029 ± 0.002^d^	10.77 ± 2.70^c^
FB	1.73 ± 0.02^d^	0.021 ± 0.004^a^	0.075 ± 0.001^a^	18.94 ± 5.89^c^
MY	2.71 ± 0.25^b^	0.004 ± 0.005^c^	0.065 ± 0.004^c^	57.04 ± 5.54^a^
FBMY	1.87 ± 0.05^c^	0.012 ± 0.003^b^	0.071 ± 0.002^b^	43.03 ± 4.19^b^

*N.D. means the detection result is below limit of quantitation, i.e., not detected.

Panels marked with different lowercase letters are significantly different (*p* < 0.05).

### Effects of *C. militaris* fermentation products on food and water intake and body weight gain

**Figure 1:**
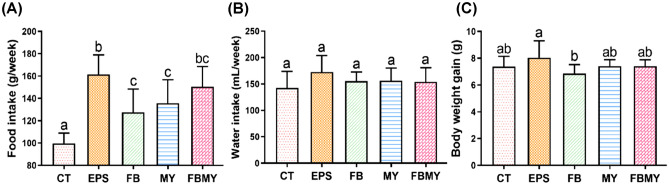
Effects of *C. militaris* fermentation products on food and water intake and body weight gain of JM mice during the 28-day experiment. Food intake **(A)**, water intake **(B)**, and body weight gain **(C)** were shown in different indicated groups. Values are presented as means ± SD (n = 5 for all test groups). Panels marked with different lowercase letters are significantly different (*p* < 0.05).

Food and water intake and body weight gain in mice of all groups are presented in Fig. 1. The food intake of JM mice in the experimental groups fed with *C. militaris* fermentation products (EPS, FB, MY, and FBMY) was significantly higher than that of the CT group that were fed a normal diet (all *p* < 0.05; Fig. 1A). JM mice in the EPS group had the highest food intake (*p* < 0.001 vs. CT, *p* = 0.007 vs. FB, *p* = 0.034 vs. MY, *p* = 0.343 vs. FBMY). A possible explanation for this phenomenon is that the sweetness of EPS increases the palatability of the pellets. Moreover, there were no significant differences in water intake (Fig. 1B) or body weight gain for 28 days (Fig. 1C) between the experimental groups and the CT group (all *p* > 0.05). Although mice in the EPS group ate more than mice in the other groups, their body weight gains were only significantly increased when compared with the FB group (*p* < 0.05). These results imply that body weight gain in JM mice is controlled or regulated by EPS addition, thereby preventing obesity.

Recently, the regulatory role of some medicinal fungi on obesity has been gradually clarified through 16S rRNA gene sequencing and metabolomic studies^17–22^. Since intestinal bacteria can not only increase the absorption of intestinal monosaccharides and short-chain fatty acids and promote the fat accumulation in the liver tissue^23^, but the lipopolysaccharide of gram-negative bacteria can also bind to receptors on the surface of immune cells, causing an inflammatory response thereby aggravating obesity^24^. Previous studies have demonstrated that *O. sinensis* can reduce the amounts of *Enterococcus cecorum* and modulate a bile acid receptor, thereby attenuating lipid metabolism disorders and their associated inflammation in high-fat diet (HFD) mice^25^. *C. militaris* polysaccharides could increase gut bacteria negatively associated with obesity traits, decrease the population of gram-negative bacteria, effectively improve intestinal flora dysbiosis in HFD-induced mice, and regulate the levels of metabolites, thereby reducing body weight, fat accumulation, and pro-inflammatory cytokine levels in mice^26,27^. Selenium-enriched *C. militaris* could reduce serum triglyceride and low-density lipoprotein cholesterol levels, inhibit serum lipopolysaccharide-binding proteins, adiponectin levels, and pro-inflammatory gene expression in HFD mice, and improve the expression of related anti-inflammatory genes^28^. Although the mechanisms remain to be elucidated, *C. militaris* fermentation products, especially EPS, may effectively prevent obesity during the development of JM mice by modulating the intestinal flora.

### Effects of *C. militaris* fermentation products on apparent testicular parameters

**Figure 2:**
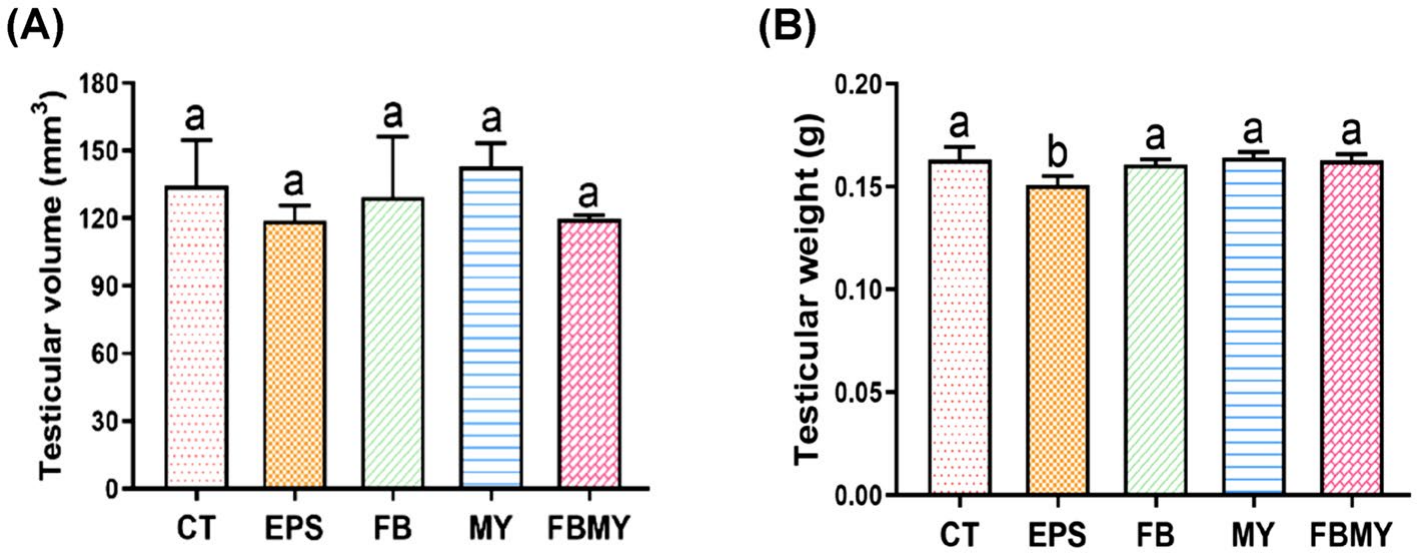
Effects of *C. militaris* fermentation products on apparent testicular parameters of JM mice. Testicular volumes **(A)** and testicular weights **(B)** were shown in different indicated groups. Values are presented as means ± SD (n = 5 for all test groups). Panels marked with different lowercase letters are significantly different (*p* < 0.05).

Testicular volumes and weights of JM mice in each group are shown in Fig. 2. There were no significant differences in testicular volume between the experimental and CT groups (all *p* > 0.05; Fig. 2A). Mice in the MY group had the largest testicular volumes. Furthermore, mice in all groups, except the EPS group, showed similar results (all *p* > 0.05; Fig. 2B). Mice in the EPS group had the lowest testicular weight and showed significant differences compared with mice in the other groups (*p* = 0.004 vs. CT; *p* = 0.013 vs. FB; *p* = 0.002 vs. MY; *p* = 0.005 vs. FBMY).

It has been reported that cordycepin reduces fat accumulation around the epididymis of HFD rats^29^ and significantly reduces epididymal adipocyte sizes in a dose-dependent manner^30^. In addition, *C. militaris* polysaccharides can decrease epididymal fat index by modulating the expression of key genes and proteins in epididymal fat^31^, including reducing sterol regulatory element binding protein-1c level by approximately 49%, inhibiting the expression of peroxisome proliferator-activated receptor γ protein by approximately 67%^32^, thereby significantly reducing epididymal adipogenesis. This suggests that the decreased testicular weight of JM mice in the EPS group may be related to the fact that EPS can reduce lipid and adipocyte differentiation in testis and surrounding tissue. Since the testes play crucial roles in producing sperms and synthesizing androgens, the testicular structure, sperm parameters, and sex hormone levels in sera were further evaluated.

### Effects of *C. militaris* fermentation products on testicular morphology and structure

**Figure 3:**
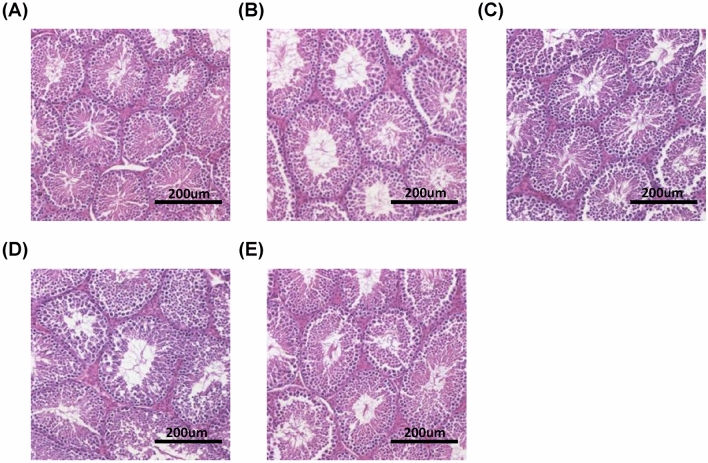
Effects of *C. militaris* fermentation products on testicular morphology and structure. Testicular sections of the CT group **(A)**, EPS group **(B)**, FB group **(C)**, MY group **(D)**, and FBMY group **(E)** were stained with H&E. Scale bars: 200 μm. Panels marked with different lowercase letters are significantly different (*p* < 0.05).

The hematoxylin and eosin (H&E) staining images of testicular sections of JM mice from each group are shown in Fig. 3. The morphology and structure of the seminiferous tubule (STs) were normal, and germ cells in the STs were in a regular arrangement in all groups. In the same field of view, STs were larger, and sperm cell densities were higher in all experimental groups than in the CT group. These results indicate that *C. militaris* fermentation products promote ST development in the testes of JM mice. Therefore, we evaluated the effect of *C. militaris* fermentation products on the morphological indicators of STs.

**Figure 4:**
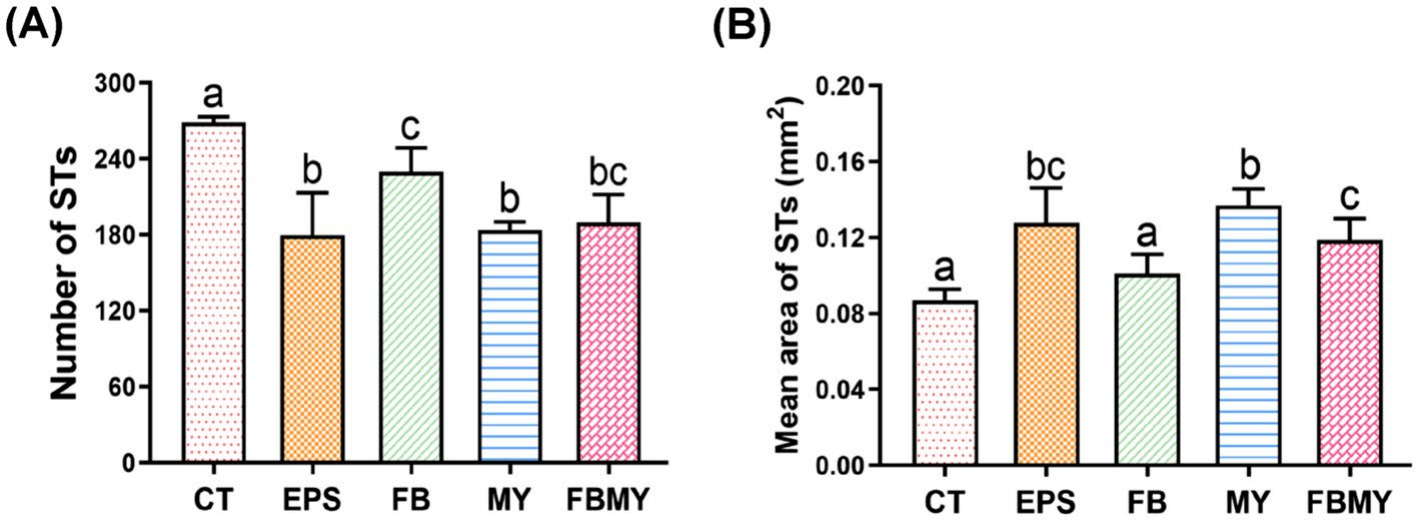
Effects of *C. militaris* fermentation products on the numbers **(A)** and mean area **(B)** of STs in testicular sections in different indicated groups. Values are presented as means ± SD (n = 5 for all test groups). Panels marked with different lowercase letters are significantly different (*p* < 0.05).

The number and mean area of the STs of JM mice in each group are presented in Fig. 4. The number of STs in all experimental groups was significantly lower than that in the CT group (*p* < 0.05; Fig. 4A). Mice in the EPS group had the lowest number of STs (*p* < 0.001 vs. CT, *p* < 0.001 vs. FB, *p* = 0.761 vs. MY, *p* = 0.052 vs. FBMY). Moreover, the mean area of STs in all experimental groups, except for the FB group, was significantly larger than that in the CT group (all *p* < 0.05; Fig. 4B). The mean area of STs was the largest in the MY group (*p* < 0.001 vs. CT, *p* = 0.221 vs. EPS, *p* < 0.001 vs. FB, *p* = 0.025 vs. FBMY). The larger the mean area of the STs, the more mature the STs.

The STs of the testis are the site of spermatogenesis and are composed of spermatogenic cells and Sertoli cells, the former producing sperm and the latter supporting and nourishing spermatogenic cells^33^. In this study, except that EPS significantly reduced testicular weight, *C. militaris* fermentation products did not significantly affect testicular volumes or weights of JM mice, but significantly increased the size and maturation of STs. These results suggest that *C. militaris* enhances testicular development, thereby promoting spermatogenesis in JM mice.

### Effects of *C. militaris* fermentation products on sperm parameters

To further assess male reproductive development, sperm concentrations and ratios of motile sperm in the epididymis were analyzed (Table 2). Sperm concentrations in JM mice in all experimental groups were higher than in the CT group. Mice in both the FB and MY groups showed significant increases in sperm concentrations compared to the CT group (*p* < 0.05). Mice in the MY group had the highest sperm concentrations. Furthermore, the ratio of motile sperm was also higher in all experimental groups than in the CT group. Except for the MY group, the motile sperm ratios in all experimental groups were significantly higher than in the CT group (all *p* < 0.05). Ratios of motile sperm were the highest in the FBMY group.

**Table 2:** Effects of *C. militaris* fermentation products on sperm parameters in epididymis of JM mice.

Group	Sperm concentrations (× 10^6^/mL)	Ratios of motile sperms (%)
CT	0.96 ± 0.16^b^	84.28 ± 2.00^a^
EPS	1.11 ± 0.13^ab^	88.91 ± 2.00^b^
FB	1.19 ± 0.08^a^	88.21 ± 3.00^b^
MY	1.28 ± 0.08^a^	86.73 ± 2.00^ab^
FBMY	1.06 ± 0.16^ab^	89.59 ± 2.00^b^

*Values are presented as means ± SD (n = 5 for all test groups). Panels marked with different lowercase letters are significantly different (*p* < 0.05).

Based on the results shown in Figs. 3, 4 and Table 2, *C. militaris* fermentation products promoted the development and maturation of STs, enhanced spermatogenesis in the testes, and increased mature sperm concentration and motile sperm ratio in the epididymis. These results are consistent with reports showing increased sperm concentrations and motilities in boars^34^ and Sprague–Dawley rats^35^ following MY supplementation.

Oxidative stress is an important cause of testicular damage, which can lead to testicular dysplasia, spermatogenic cell necrosis and apoptosis, resulting in male reproductive dysfunction^36^. *C. militaris* is a natural antioxidant with excellent ability to scavenge reactive oxygen radicals^37^. Among them, cordycepin can regulate the activities of downstream antioxidant enzymes through the SIRT1/Foxo3a signaling pathway^38^, as well as regulate NF-κB activation and MAPKs signaling^39^, effectively improving sexual dysfunction in aged rats. Moreover, another study showed that the EPS extracted from the *C. militaris* FB could promote the proliferation of spleen T and B lymphocytes through MAPK signaling^40^; thus, EPS may also be able to resist testicular oxidation through this pathway. Therefore, the antioxidant activities of *C. militaris* may explain why these fermented products can promote male reproductive development in JM mice.

### Effects of *C. militaris* fermentation products on serum sex hormone levels

**Figure 5:**
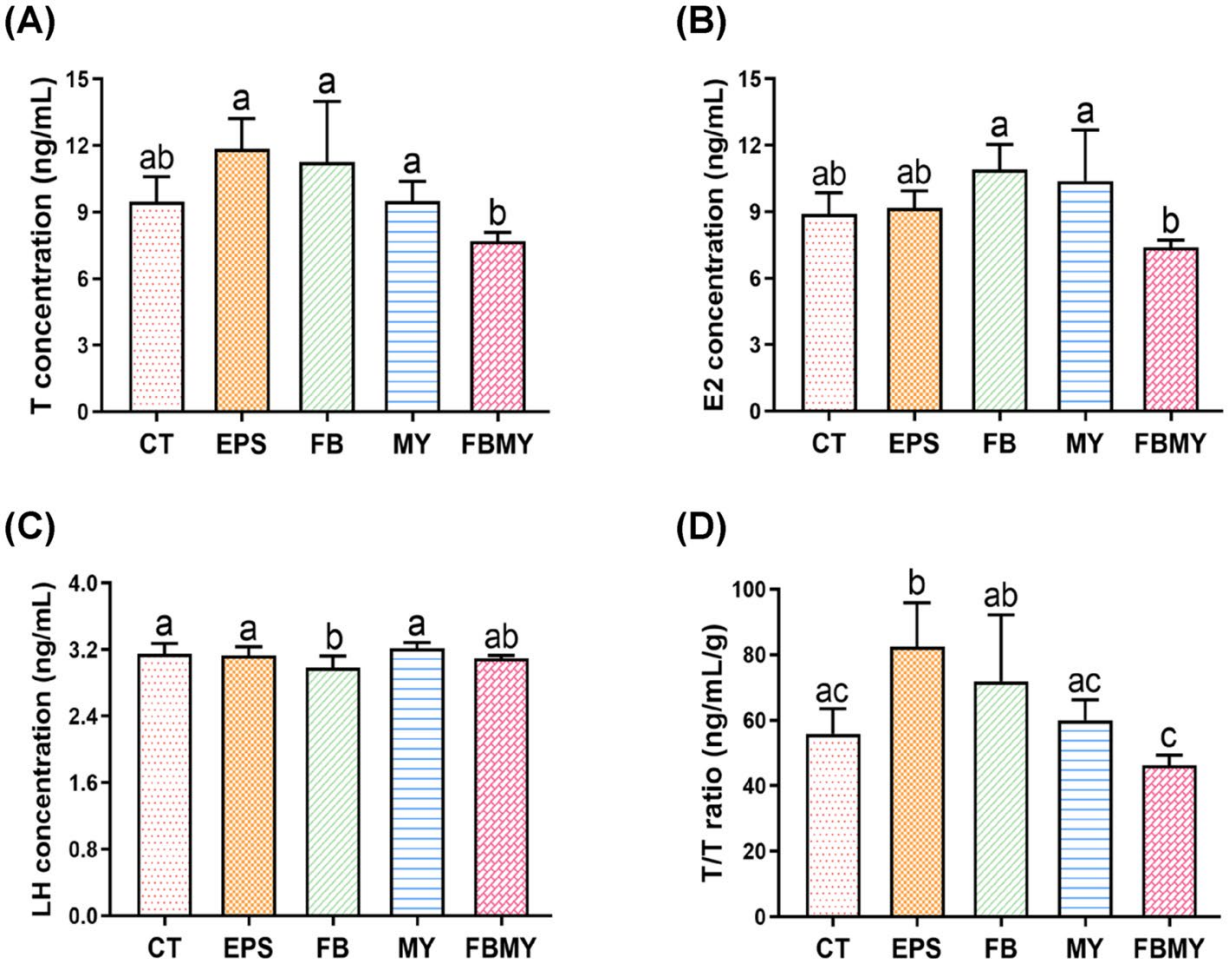
Effects of *C. militaris* fermentation products on serum sex hormone levels in different indicated groups. T concentration **(A)**, E2 concentration **(B)**, LH concentration **(C)**, and calculated T/T ratio **(D)** were shown. Values are presented as means ± SD (n = 5 for all test groups). Panels marked with different lowercase letters are significantly different (*p* < 0.05).

To investigate the effects of supplementation with *C. militaris* fermentation products on male reproductive development, the serum sex hormone levels of JM mice were analyzed (Fig. 5). Although mice in both the EPS and FB groups showed higher testosterone (T) concentrations than those in the CT group, there were no significant differences in T concentrations between each experimental group and the CT group (all *p* > 0.05; Fig. 5A). Mice in the EPS group had the highest T levels. However, T levels in mice in the FBMY group were significantly lower than those in the other experimental groups (all *p* < 0.05), but not in the CT group.

Since estradiol (E2) levels may antagonize the effects of T, serum E2 levels were also analyzed. Although mice in both the FB and MY groups showed higher E2 concentrations in sera than those in the CT group, there were no significant differences in E2 concentrations between each experimental group and the CT group (all *p* > 0.05; Fig. 5B). However, E2 levels were the lowest in the FBMY group but only significantly different when compared to the FB and MY groups (both *p* < 0.05). In males, E2 is mostly converted through the aromatization of T^41^. The results shown in Figs. 5A,B support the positive correlations between T and E2 levels, and the lower E2 concentration, resulting in lower testicular volumes, number of STs, and sperm concentrations in epididymis of mice in the FBMY group. However, the reason for the lower T and E2 levels in the FBMY group than in the other groups remains to be elucidated.

Since luteinizing hormone (LH) is secreted by the anterior pituitary gland, which stimulates ovulation in females and androgen synthesis in males, serum LH levels were analyzed. Except for the FB group, there were no significant differences in LH concentrations among the other groups (all *p* > 0.05; Fig. 5C). However, the LH levels were significantly lower in the FB group than in the CT, EPS, and MY groups (all *p* < 0.05). The synthesis and secretion of sex hormones are regulated mainly by the hypothalamus-pituitary-gonadal axis. Thus, increases in both T and E2 levels in the FB group may have a negative effect on the LH levels secreted by the pituitary gland. This explains why mice in the FB group had the lowest LH levels.

Since T concentrations in testes are at least 25–100 times higher than those in sera^42^, the ratios of serum T levels to testicular weights (T/T) were calculated to directly assess the ability of testes to synthesize T. Results showed that T/T ratios were higher in all experimental groups, except the FBMY group, than those in the CT group (Fig. 5D). The T/T ratios were the highest in the EPS group and showed significant differences when compared with the CT, MY, and FBMY groups (all *p* < 0.05). Moreover, the T/T ratios of the FBMY group were the lowest and showed a significant decreases when compared with those of the EPS and FB groups ( *p* < 0.05), but there was no significant difference with the CT group.

The results shown in Fig. 5 indicated that *C. militaris* fermentation products, especially EPS, promote the development of testes and elevate serum levels of T and T/T ratios in JM mice. Our findings agree with one previous study that demonstrated a *C. militaris*-induced increase in T productions in Sprague-Dawley rats ^43^. Another study showed that *O. sinensis* stimulates T productions in vivo and in vitro ^44^. Polysaccharides and glycoproteins of *O. sinensis* share structural similarities with LH and can be recognized by LH receptors on Leydig cells to stimulate T productions. Although the specific mechanism remains unknown, *C. militaris* fermentation products, especially EPS, showed androgen-like activity, which can promote male reproductive development in JM mice.

The results shown in Figs. 2, 3, 4 and 5 and Table 2 indicate that *C. militaris* fermentation products, especially EPS, promote the development of testes, elevate serum levels of T and T/T ratios, and then increase sperm concentrations and ratios of motile sperms in the epididymis of JM mice. Since all mice in this study were fed ad libitum, it is possible that the EPS-containing feed increased the mice's appetite due to its sweetness. From our results, increased EPS intake may better promote reproductive development in male JM mice. In addition, *C. militaris* extract has been reported to prevent obesity and fat accumulation in ovariectomized rats, lower triglyceride levels, and enhance estrogen receptor agonistic activity and phosphorylation, effectively regulating menopause-induced obesity^45^. Therefore, these findings suggest that *C. militaris* fermentation products can promote the reproductive system health and prevent metabolic syndromes including obesity.

## Conclusion

In this study, we evaluated the effects of four different *C. militaris* fermentation products on male reproductive development in JM mice. EPS showed the strongest androgen-like activity, which promoted male reproductive development. EPS supplementation can increase the mean area and maturation of STs in the testes, sperm concentrations and ratios of motile sperms in the epididymis, and serum T levels and T/T ratios. Therefore, EPS supplementation can effectively improve male reproductive development in JM mice, and may prevent or assist in treating male reproductive dysfunction or infertility. Furthermore, it is important to note that *C. militaris* fermentation products can effectively control or regulate body weight gain in JM mice during the experimental period of 28 days, thus it has the potential to assist in obesity treatment. However, it must be noted that clinical studies are required to determine if these beneficial effects of *C. militaris* can apply to humans.

## Materials and methods

### Materials

*C. militaris* fermentation products including EPS, FB, and MY, were gifts from Dalong Biotechnology Co. Ltd. (Taichung, Taiwan). EPS were obtained from ethanol precipitation of the FB, centrifugation, and freeze-drying. The yields of polysaccharides, cordycepin, adenosine, and proteins of the EPS, FB, MY and whole fermentation product (FBMY) were detected by the phenol-sulfuric acid method ^46^ high-performance liquid chromatography (HPLC) method ^47^, and Bio-Rad Protein Assay (Bio-Rad, Hercules, CA, USA) according to the manufacturer's instructions, respectively (Table 1). The feed pellets of different experimental groups were produced by mixing 5% (w/w) dry weight of EPS, FB, MY, and FBMY with 95% Laboratory Rodent Diet 5001.

### Animals

Three-week-old male mice (C57BL/6JNarl), 25 in number, were purchased from the National Laboratory Animal Center (Taipei, Taiwan). During the experimental period, they were kept in an air-conditioned room at 24 ± 2 °C and 50–60% humidity with a 12 h light/dark cycle. They were randomly divided into five groups (n = 5 each). Mice in the control (CT) group were fed Laboratory Rodent Diet 5001 only, while mice in the experimental groups were fed pellets containing 5% (w/w) EPS, FB, MY, and FBMY for 28 days. Mice were sacrificed using the cervical dislocation method after anesthesia with isoflurane (Baxter, CA, USA) at the end of the experiment. Blood samples were obtained by cardiac puncture. In addition, testes and epididymides were removed for measurement and analysis.

### H&E staining of testis sections

Each right testis was fixed in a 10% neutral formalin solution (Leica Surgipath Inc., Buffalo Grove, IL, USA) for more than 16 h. After fixation, testes were cut in half at the widest point and embedded in paraffin. Five-micrometer histological sections were deparaffinized, rehydrated, and stained with H&E according to the manufacturer’s instructions (Sigma-Aldrich Corp., St. Louis, USA). Morphological images were obtained using a TissueFAXS system (Tissue Gnostics, Vienna, Austria) at the Instrument Center of Chung Shan Medical University. Seminiferous tubule (ST) numbers were recorded using Scope Photo 3.0 (Micro Imaging Ltd., Auckland, New Zealand).

### Sperm concentrations and motilities

The right epididymis was cut into three pieces and incubated in 1 mL of Ren’s solution at 37 °C for 30 min. After centrifugation at 100 g for 5 min, sperm concentrations were determined using a hemocytometer and Motic BA300 Binocular Compound Microscope (Motic Asia, Hong Kong, China). For each mouse, sperm numbers were obtained for five grids and then divided by five. Sperm concentrations were expressed in millions per milliliter (10^6^ cells/mL), and sperm images were captured at 5 s intervals using a Dino-Lite digital microscope (AnMo Electronics Corp., Taipei, Taiwan) and DinoCapture 2.0. Active sperms were defined as sperm in different positions on the two images. Ratios of motile sperm (%) = (number of total sperm–number of inactive sperm)/number of total sperm × 100%.

### Serum hormone assays

The collected blood samples were centrifuged at 10,000 g for 10 min, and serum was collected. Serum testosterone (T), estradiol (E2), and luteinizing hormone (LH) levels were determined according to the manufacturer’s protocols (Elabscience Biotechnology Co., Ltd., Wuhan, Hubei, China).

### Statistics

Data are expressed as mean ± standard deviation (SD). The differences between multiple groups were evaluated using one-way analysis of variance (ANOVA) and post-hoc least significant difference (LSD) tests. Statistical significance was set at *p* < 0.05. All figures were created using Graph Pad Prism version 6.0.0 for Windows (Graph Pad Software, www.graphpad.com).

### Ethical approval

Experimental protocols were approved by the Animal Care and Use Committee of Da-Yeh University (approval number: 107025), all animals were treated according to the guidelines for animal experimentation of Da-Yeh University in Changhua, Taiwan. The animal experiments were also performed in accordance with the ARRIVE (Animal Research: Reporting of In Vivo Experiments) guidelines.

## Data Availability

The datasets used and/or analysed during the current study available from the corresponding author on reasonable request.
